# Tryptophan Metabolites at the Crossroad of Immune-Cell Interaction via the Aryl Hydrocarbon Receptor: Implications for Tumor Immunotherapy

**DOI:** 10.3390/ijms22094644

**Published:** 2021-04-28

**Authors:** Marco Gargaro, Giorgia Manni, Giulia Scalisi, Paolo Puccetti, Francesca Fallarino

**Affiliations:** Department of Medicine and Surgery, University of Perugia, 06132 Perugia, Italy; manni.giorgia87@gmail.com (G.M.); giulia.scalisi@gmail.com (G.S.); paolo.puccetti@unipg.it (P.P.)

**Keywords:** aryl hydrocarbon receptor, cancer, gut metabolites, dendritic cells, immunotherapy

## Abstract

The Aryl hydrocarbon receptor (AhR) is a critical regulator of both innate and adaptive immune responses, with potent immunomodulatory effects that makes this receptor an attractive molecular target for novel therapeutics. Accumulating evidence indicates that diverse—both host’s and microbial—tryptophan metabolites profoundly regulate the immune system in the host via AhR, promoting either tolerance or immunity, largely as a function of the qualitative and quantitative nature of the metabolites being contributed by either source. Additional findings indicate that host and microbiota-derived tryptophan metabolic pathways can influence the outcome of immune responses to tumors. Here, we review recent studies on the role and modalities of AhR activation by various ligands, derived from either host-cell or microbial-cell tryptophan metabolic pathways, in the regulation of immune responses. Moreover, we highlight potential implications of those ligands and pathways in tumor immunotherapy, with particular relevance to checkpoint-blockade immune intervention strategies.

## 1. Introduction

The aryl hydrocarbon receptor (AhR) is a ligand-activated transcription factor that is activated by small molecules provided by microorganisms, the diet, metabolism, and pollutants. Several studies have shown that AhR signaling plays important roles in the immune system, both in health and disease. AhR is expressed by several immune cells, and it is emerging as a central and molecular node capable of integrating the effects of xenobiotic and microbial molecules as well as host-cell derived metabolites during immune responses [1,2]. As small molecules regulate its activity, AhR represents a potential target for therapeutic immunomodulation. Widely expressed in a variety of animal species as well as in humans, AhR has constitutive functions that have only recently begun to be appreciated. Animal and human data indicate that AhR is involved in various signaling pathways critical to cell normal homeostasis, which covers multiple aspects of physiology, such as cell proliferation, differentiation, gene regulation, cell motility, migration, inflammation and others [3,4]. Among those, regulation of the immune response appears to be one of the main functions of AhR [5,6].

A connection between AhR and immune system has long been recognized [7]. Accordingly, studies using AhR-deficient mice have shown that impaired AhR activity correlates with diminished levels of IL-22–producing ILC type 3 (ILC3) and consequently culminates in a worsening of inflammatory diseases, such as colitis [2]. Moreover, it has been shown that certain tumors can escape otherwise protective immune recognition and consequent growth control via immunosuppressive AhR signaling pathways, activated by specific metabolites produced by host cells or microbiota [6,8,9].

Previous studies demonstrated a crucial role of AhR in adaptive immune responses for both T(H)17 and T(reg) and cell differentiation after AhR activation by specific ligands pointing as crucial target for therapeutic immunomodulation [10]. Moreover, it is now emerging that the AhR expression is modulated during the course of activation and differentiation of several immune cells. Accordingly numerous studies have reported the expression of AhR in several immune cell types including B cells, mucosal cells, and antigen-presenting cells (APCs) [11]. In T cells, AhR is more expressed in mouse T helper type 17 (Th17) cells as compared to non-polarized Th0 cells as well as to Th1, Th2, and regulatory T (Treg) cells [12].

Overall, these pieces of evidence make AhR-expressing immune cells attractive cellular targets, whereby AhR interference would represent an important means of tipping the immune balance in favor of immunity, as it would desirable in tumor immunotherapy.

Studies on various tumor types and tumor cell lines have shown high AhR expression, suggesting that AhR is activated constitutively by specific ligands in tumors and facilitates their growth or immune evasion [13]. Specifically, in tumor cells, emerging evidence suggests a promoting role for AhR in the initiation, promotion, progression, invasion, and metastasis of cancer cells [1,5,14,15]. The identification of potential endogenous ligands—particularly, those derived by tryptophan (Trp) and indole metabolism, able to regulate AhR activity—opens the possibility of designing ad hoc molecules with pharmacological and/or therapeutic value to treat human diseases in which AhR may have an important role [3]. However, an emerging concept that might be of value in the search for therapeutic AhR ligands is the inadequacy of the ‘agonist vs. antagonist’ classification of those ligands [16,17], as well as the occurrence of novel, alternative ligand-specific signaling pathways for this receptor [18,19,20,21]. Thus there are both potential opportunities and challenges of developing AhR-centered therapeutics [6].

Here we review the multiple Trp metabolic pathways able to generate AhR ligands and highlight potential implications of those pathways and produced metabolites in tumor immunotherapy strategies.

## 2. AhR Ligands: From the Discovery to the Main Emerging Role of Trp Derivatives in Immune Regulation

The AhR has been discovered in an effort to uncover the mechanisms responsible for 2,3,7,8-Tetrachlorodibenzodioxin TCDD toxicity [22], as accurately reviewed elsewhere [4]. In several industrial incidents, a severe skin disease, termed chloracne, was observed in exposed workers and human populations. Suspected chemicals were tested for chloracne-like symptoms on the rabbit ear, and TCDD was identified as the most potent agent producing chloracne. In 1992, Christopher Bradfield’s and Fuji-Kuriyama’s groups independently identified the AhR as a ligand-activated transcription factor of the PAS (Per-Arnt-Sim) family [23,24]. Then, the cDNA encoding a human Ah receptor, mediating the toxic effects of TCDD was identified in 1993, and it was found to be expressed at its highest levels in placenta, lung and heart [25].

**Figure 1:**
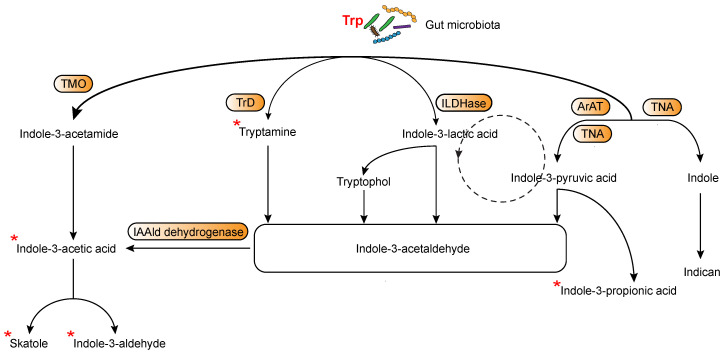
Main routes of tryptophan metabolism in microbiota. TMO, Tryptophan 2-monoxygenase, TrD, Tryptophan decarboxylase, ILDHase, Indole-3-lactic acid dehydrogenase, ArAT, Aromatic amino acid aminotransferases, TNA, Tryptophanase, IAAld (Indole-3-acetaldehyde) dehydrogenase. * Indicates known AhR ligands.

**Figure 2:**
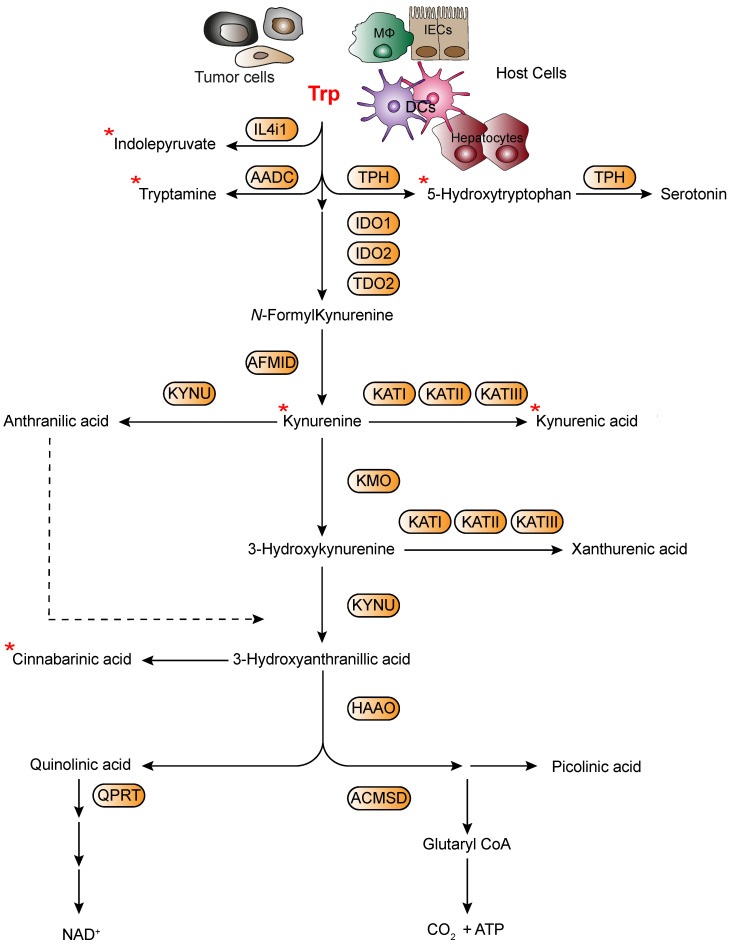
Main routes of tryptophan metabolism in host’s cells. AADC, aromatic-l-amino acid decarboxylase, TPH, tryptophan hydroxylase, IDO1, indoleamine-2,3-dioxygenase 1, IDO2, indoleamine-2,3-dioxygenase2, TDO, tryptophan-2,3-dioxygenase, AFMID, kynurenine formamidase, KATs, kynurenine amino transferases I–III, KMO, kynurenine 3-monooxygenase, KYNU, kynureninase, HAAO, 3-hydroxyanthranilate 3,4-dioxygenase, ACMSD, α-amino-β-carboxymuconate-ε- semialdehyde decarboxylase, QPRT, quinolinic acid phosphoribosyl transferase. * Indicates known AhR ligands.

In more recent years, the relationship between immune responses and AhR has particularly attracted the attention of many researchers and it was clearly shown that AhR can be considered one of the important players involved in orchestrating host–microbe homeostasis [2,26]. In fact, several studies have highlighted that the gut microbiota metabolism produces several ligands of AhR, thereby activating specific immunoregulatory pathways [27,28]. Among those ligands, accumulating evidence demonstrates that microbiota may affect the host immune system by modulating Trp metabolism [29]. Yet, intestinal bacteria, such as *Lactobacillus reuteri* can also directly utilize Trp to produce many immunologically important metabolites with indole, indolic acid derivatives and tryptamines being the main microbial tryptophan metabolites in the gut [2,30]. Notably, indole and indolic acid derivatives can be further metabolized into other final products, such as the conversion of indole-3-acetate to skatole or indole-3-aldehyde, indole to indicant, and indole acrylic acid to indole propionic acid additionally acting as AhR ligands [31,32] (Figure 1). In addition to microbial-derived tryptophan metabolites, cells in the liver and extrahepatic tissues can generate additional tryptophan metabolites by means of Trp not being used in protein synthesis. Specifically, Trp can be metabolized through three main metabolic pathways, namely the kynurenine pathway (KP) [33], the serotonin [34] and the interleukin 4 induced 1 pathway [35] (Figure 2). Approximately 95% of the Trp ingested is degraded to l-kynurenine, kynurenic acid (KA), quinolinic acid, picolinic acid, and nicotinamide adenine dinucleotide (NAD) via the KP, which is regulated by three rate-limited enzymes: tryptophan 2,3-dioxygenase 2 (TDO2) in the liver and indoleamine 2,3-dioxygenases (IDO1 and IDO2) in extrahepatic tissues [29,36]. Among Trp metabolites, either produced from host’s cells or microbiota, several have the ability to bind and activate AhR, including l-kynurenine, kynurenic acid, xanthurenic acid, and cinnabarinic acid from host’s cells or indole, indole propionic acid, indole acetic acid, skatole, indole-3-carboxaldehyde, and tryptamine from microbiota (Figure 1 and Figure 2). In addition to those listed above, additional Trp/indole-related metabolites have been described as additional potential endogenous AhR ligands; these include FICZ (6-formylindolo[3,2-b]carbazole; an ultraviolet photoproduct of tryptophan), indirubin and indigo [37,38].

Notably, several studies have reported that host and bacterial Trp metabolites may have profound effects on the regulation of the host’s immune system and immune system-microbiota interaction via AhR, [27,28,39], suggesting the potential for tackling these pathways in novel immunotherapy approaches.

## 3. Binding Mode of Ligands to AhR: An Aspect Influencing Canonical Categorization of Agonism and Antagonism?

Molecules functioning as ligands for AhR activate AhR heterodimers at promoter recognition sequences of the target genes. The AhR/AhR nuclear translocator (ARNT) complex may then require coactivators (including members of other families of transcription factors [40]) in order to initiate transcription, and to unwind histone-bound DNA for exposing additional promoter recognition sites via their histone acetyltransferase function. Within this scenario, at least four major factors appear to contribute to the outcome of gene transcriptional regulation by AhR, namely, the nature of the ligand, its capacity to be further metabolized by AhR-induced enzymes, the local tissue microenvironment, and the presence of coactivators in the cell. Prototypical examples in this sense are represented by AhR activation by three different Trp derived metabolites in gut innate lymphoid cells by microbiota-derived indole-3-aldehyde (IAld) [30], in skin keratinocytes by endogenous FICZ [41] and in lymphoid-tissue dendritic cells by Kynurenine [42].

Studies combining homology modeling, docking analyses, molecular dynamic simulations with mutagenesis experiments and gene profiling reported that TCDD and two different Trp metabolites—namely, Kynurenine and FICZ—are able to bind mouse AhR by exploiting different key interactions with distinct sets of fingerprint residues [19]. As a result, they potentially stabilize different conformations of AhR that, in turn, selectively regulate downstream signaling pathways and transcription of specific target genes. This is in line with previous observations that the AhR fingerprint residues required for activation by dioxin are distinct from those necessary for activation by kynurenine, even when the response being measured is the same, namely, transcription of a gene (*Cyp1a1*), whose promoter contains AhR-specific xenobiotic response elements. A mutated form of the receptors that does not bind kynurenine will, instead, bind dioxin with increased potency and likely affinity [16]. Collectively, these results suggest that, in determining the qualitative effect of AhR engagement, it is not the potency (dictated in turn by affinity) and the efficacy of the ligand that matter so much, as the ligand’s ability to select a specific conformation of the receptor [42]. When contextualized to the widely accepted conformation-based operational model of agonism (which considers multiple active receptor conformations, agonist efficacy and maximum effect of the system), it is likely that different AhR ligands preferentially bind distinct conformations of the AhR complex—each having a distinct set of fingerprint residues—thus initiating different pathways of downstream signaling and transcriptional events.

Altogether, these data cast new light on the canonical categorization of AhR ligands as ‘agonists’ or ‘antagonists’ [18,20,21] and, at the same time, they open new avenues for the design and development of selective AhR modulators that, by targeting specific receptor conformations associated with specific AhR functions, may offer novel therapeutic opportunities in specific diseases that may be associated with the receptor [17,19].

## 4. The Various Trp Metabolic Pathways and Metabolites in the Regulation of Immune Responses to Tumor Cells via AhR Activation

### 4.1. Tumor Microenvironment-Derived Trp Metabolites

It is now clear that most tumors are complex ecosystems that emerge and evolve under robust selective pressure from their microenvironment (TME), which involves immunological, trophic, metabolic, and therapeutic factors. Such pressure promotes the differentiation of both malignant and nonmalignant (i.e., endothelial, stromal, and immune) cells of the TME, culminating in disparate degrees of intratumoral heterogeneity, and resulting in disease progression and resistance to specific treatment [43,44]. Progressing tumors tend to acquire driver mutations that favor some degree of genetic instability, and immunoescape (for example, loss of major histocompatibility complex (MHC) class I-coding genes or capacity to release suppressive cytokines) [45].

Cells of the TME such as tumor-infiltrating lymphocytes (TILs) [46,47], tumor-associated fibroblasts [48] as well as various myeloid cell populations, such as tumor-associated macrophage and dendritic cells [49], can acquire suppressive functions in the TME. These complex cell networks in the TME influence immune cell functions within the tumor, depending on the communication between immune cells and other tumor-associated cells [49]. Many metabolites, and metabolic enzymes are immunosuppressive within the TME and directly affect T- and other immune-cell functions. Especially, when T cells lose the competition with highly metabolically active tumor cells for access to nutrients, their functional capacity is diminished [50].

Disturbance in Trp metabolism and/or AhR activation is strongly associated with several tumors, pointing to Trp metabolite/AhR signaling modulation as an interesting therapeutic perspective. Notably, upregulation of IDO1 or TDO2 enzymes by tumor cells, stromal cells and/or mononuclear phagocytes in the TME results in activation of Trp catabolism, depriving T cells of the essential amino acid Trp, and, at the same time, generating Trp metabolites that are toxic to T cell responses [51] or are able to induce Treg-cell differentiation or immunosuppressive function of immature myeloid cells [52]. New studies clearly highlight that in addition to classical and well-known pathways of Trp metabolism, such as those involving IDO1 and TDO2, Trp can be metabolized by alternative routes, leading to the generation of biologically active metabolites that are also potent AhR ligands [30,33]. This fact may explain why selective blockade of solely IDO1 pathway may have failed in clinical trials [53] and may have not been sufficient to efficiently reprogram the TME for immune activation. Moreover, failure of these trials might be related to the lack of information about IDO1 expression and activity (kynurenine production) at the tumor site or systemically in the patients enrolled in the studies.

Notably, a recent publication has shown that an active IDO/TDO2-Kyn-AhR pathway associates with immune suppressive features in human tumors and that AhR blockade will reverse IDO/TDO2-mediated immunosuppression [54]. Because the immunoregulatory Trp metabolite kynurenine can be produced both by IDO1 and TDO2, additional approaches may involve the development of dual inhibitors of both enzymes. CMG017 and CB548, two dual inhibitors of IDO1 and TDO2, have been shown to potently suppress the kynurenine pathway and they showed promising anti-tumor efficacy, with favorable pharmacologic profiles, overcoming resistance to immune checkpoint inhibitors [55]. Another elegant, alternative approach involves kynurenine depletion with a therapeutic enzyme. Specifically, administration of a recombinant bacterial enzyme, kynureninase (KYNase), able to degrade kynurenine, has been shown to produce substantial therapeutic effects when combined with approved checkpoint inhibitors or with a tumor vaccine for the treatment of different types of experimental tumors, such as B16-F10 melanoma, 4T1 breast carcinoma or CT26 colon carcinoma tumors [56]. Specifically, PEG-KYNase resulted in prolonged depletion of Kynurenine and reversed the modulatory effects of IDO1/TDO2 upregulation in the TME. Accordingly, a recent study has shown that kynurenine controls tumor associated macrophage (TAM) activation via AhR signaling, leading to CD39 expression in TAMs and impairing the T cell response to glioblastoma tumor cells [57].

The landscape of Trp metabolites able to activate AhR, produced either by host cells or microbiota, is rapidly increasing and they involve additional pathways, other than conventional/classical IDO1/TDO2-mediated pathways. Indeed, it has been recently reported that Trp catabolism by the L-amino acid oxidase (IL4I1) elicits major effects on immunity to tumors by signaling mediated by the Trp metabolite indol-3-pyruvate (I3P) and the AhR axis. Until now, IL4I1 has mainly been implicated in immune regulatory functions that have been attributed either to the depletion of selected amino acids or to the formation of the cytotoxic molecule H_2_O_2_ [58]. A recent study identified IL4I1 as a hitherto unknown endogenous source of I3P and its downstream metabolites IAA, I3A, and ILA in humans [59] that until now, have been attributed to microbial metabolism [60]. Surprisingly, these recent results showed that I3P significantly induced AhR nuclear translocation and transcription, enhanced the motility of glioblastoma tumor cells and reduced CD8^+^ T cells proliferation in an AhR-dependent manner [59]. Compared with the established endogenous Trp AhR agonist kynurenine, I3P induced AhR activity at lower concentrations, which means that I3P represents a novel onco-metabolite. Moreover, I3P led to the production of additional AhR ligands such as kynurenic acid and indol-3-acetic metabolites that instead have been previously attributed to microbial metabolism leading to sustained AhR activation [26,30]. Interestingly, more recent observations have demonstrated that specific cytokines can induce additional Trp metabolic pathways, suppressing immune responses to tumors. Specifically, it was reported that persistently high levels of IL-2 production in TME lead to the long-lasting activation of signal transducer and activator of transcription 5, STAT5, in CD8^+^ T cells, which in turn induced strong expression of tryptophan hydroxylase 1 (5-HTP), thus catalyzing the conversion of Trp into 5-hydroxytryptophan. AhR activated by 5-HTP directly induced tumor-specific CD8^+^ TILs cell exhaustion in vivo, causing a coordinated upregulation of inhibitory receptors, such as PD-1, LAG-3, CD39 and downregulation of cytokines, thereby causing dysfunctional T cells in the TME [61]. This study clearly highlights that IL-2, by virtue of activation of a novel STAT5–5-HTP–AhR axis, induced CD8^+^ T cell exhaustion in the TME. The study reported that, this molecular pathway is not only present in mouse tumor models but is also observed in people with tumors, identifying IL-2 as a novel inducer of T cell exhaustion.

### 4.2. Microbially-Derived Trp Metabolites

**Figure 3:**
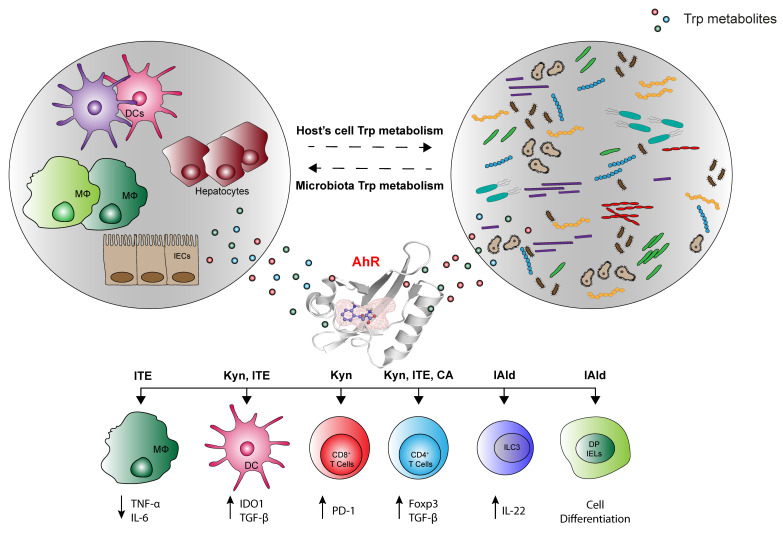
Tryptophan metabolites derived from host’s immune cells and microbiota can influence immune cell functions. Tryptophan metabolites derived from commensal bacteria and host’ cells have a critical role in modulating the homeostasis and function of innate and adaptive immune cells through indirect and direct mechanisms [71,72]. Tryptophan metabolite can activate signal transduction pathways and transcriptional programs that control the differentiation, proliferation, maturation and effector functions of many cells through activation of AhR. AhR is expressed in immune and non-immune cell types, such as intestinal epithelial cells (IECs) [73], macrophages (MΦ) [74], dendritic cells (DCs) [75], T cells [76], double- positive intraepithelial lymphocytes (DP IELs) [77], and innate lymphoid cells (ILCs) [32]. Molecules produced by host’s immune cells and microbiota can activate AhR and influence their cytokines production, cell differentiation and function (ITE: 2-(1′H-indole-3′-carbonyl)-thiazole-4-carboxylic acid methyl ester; Kynurenine (Kyn); Cinnabarinic acid (CA); Indole-3-aldehyde (IAld)).

Considering recent reports on the control of CNS resident cells by AhR ligands provided via diet and gut flora [62], it is possible to hypothesize that AhR signaling in cells in the TME may depend on the potential effects of diet, the commensal flora or other environmental factors. Indeed, dietary tryptophan can be metabolized by the gut microbiota into AhR agonists that have an effect on astrocytes to limit CNS inflammation [62]. The crosstalk between microbiota and the immune system (Figure 3), especially at the level of the gut, is extensive and critical, and it does not only promotes tolerance to commensal bacteria and oral food antigens, but also stimulates immune cells to recognize and attack opportunistic bacteria, thereby preventing bacterial invasion and infection [63,64]. These studies reported that the list of AhR ligands encopasses components of bacterial virulence factors. AhR binds bacterial pigments comprising a redox-cycling phenazine/naphthoquinone moiety, namely, *P. aeruginosa Pyo* thus leading to regulation of inflammatory leukocyte recruitment to the infected lung and control of bacterial replication [63,64].

Remarkable progress in large-scale sequencing and mass spectrometry has increased our understanding of the influence of the microbiome and/or its metabolites on the onset and progression of extraintestinal tumors and the efficacy of immunotherapy to tumors [65]. Microbiota can represent the sources of additional Trp metabolites that influence anti-tumor immunity. Recent studies have shown that particularly intestinal microbiota profoundly impacts responses of patients with specific tumors to immune-checkpoint blockade therapy [66,67]. This effect mainly arose from the enhancement of dendritic cell effector functions, thereby improving the tumor-specific CD8^+^ T cell activity [68]. The high heterogeneity of the responses to immune checkpoint inhibitor therapy in patients with tumors can be partially explained by differences in the composition of gut microbiome, with compelling evidence suggesting that specific key bacterial taxa may potentially contribute to inter-individual variation in therapeutic efficacy in clinical cohorts [66,67,69]. In this context, there is a large body of evidence that microbial metabolites derived from ingested nutrients, such as microbial Trp catabolites and short-chain fatty acids (SCFAs), are pivotal inducers of such effects [62]. However, in-depth molecular mechanisms remain as yet unclear, and research on the regulation of host-microbe interactions by these metabolites, including those derived from Trp metabolism in immune response to tumors, is still needed. Moreover, small molecule metabolites, such as indoles, also act as signaling molecules for inter-bacterial communication and quorum sensing, thereby driving changes in the function and composition of the microbiota itself to modulate intestinal homeostasis and protective immune responses in cells expressing AhR [70]. Interestingly, recent results suggest that AhR and its interacting ligands are involved in such mechanisms that may be relevant to tumor immunotherapy [64].

**Figure 4:**
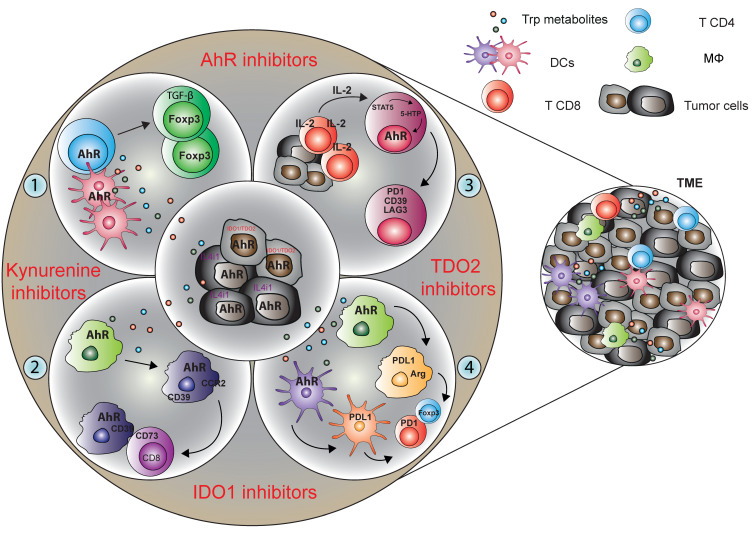
Targeting of tryptophan metabolic pathways for cancer therapy. Tryptophan-derived metabolites are immunosuppressive within the TME and directly affect T and other immune cell functions. (1) Kynurenine derived by IDO1/TDO2 competent tumor cells can affect the T cell function promoting directly or through the tolerogenic dendritic cells the regulatory T cells differentiation. (2) Moreover, kynurenine produced by glioblastoma cells activates AhR in TAMs to modulate their function and T cell immunity. AhR promotes CCR2 and CD39 expression in TAMs thus leading to CD8^+^ T cell dysfunction by producing adenosine in cooperation with CD73. (3) Molecules, such as cytokines, produced by host’s immune cells can activate AhR promoting the T cell exhaustion. In particular, high level of IL-2 leads to the persistent activation of STAT5 in CD8^+^ T cells, which in turn induces strong expression of tryptophan hydroxylase 1, thus catalyzing the conversion to tryptophan to 5-HTP. 5-HTP subsequently activates AhR nuclear translocation, causing a coordinated upregulation of inhibitory receptors and downregulation of cytokine and effector-molecule production, thereby rendering T cells dysfunctional in the tumor microenvironment. (4) In addition, IDO-Kyn-AhR-mediated immunosuppression depends on an interplay between Tregs and tumor-associated macrophages, which can be reversed by AhR inhibition. In the outer part of the circle, proposed targeting startegies able to inhibit the activity of the tryptophan metabolites are indicated in red.

These findings describe the long-distance regulation of immune cells in extraintestinal organs by gut microbiome-derived Trp metabolite signaling through AhR, opening new avenues for exploiting AhR functioning in tumor immunotherapy (Figure 4). Moreover, given the plasticity in microbial composition and function, microbial-based therapeutic interventions, prebiotics, and probiotics, as well as fecal microbial transplantation, may potentially permit the development of novel strategies for tumor immunotherapy to improve clinical outcomes.

Overall, these data provide a strong rationale for assessing active Trp metabolic pathways in tumors, for the design of combination strategies tackling AhR or specific Trp metabolites or their pathways in future clinical studies.

## 5. Conclusions

Trp metabolism through the KP and serotonin pathway and more recently though commensal microbiota metabolism largely acts via triggering of AhR-dependent signaling events in a variety of host cells (Table 1). Imbalances in Trp metabolism in disorders such as tumors have stimulated interest in therapeutically targeting the KP, particularly the main rate-limiting enzymes IDO1, IDO2 and TDO2 [78]. However, although small-molecule IDO1 inhibitors that showed promise in early-stage tumor immunotherapy clinical trials, were not effective in a phase III trial [33]. The production of immunoregulatory metabolites by additional Trp degrading enzymes, both by host cells and by specific microbiota strains, leading to AhR activaction could represent a possible reason of this failure.

Novel Trp-AhR pathway inhibitors, such as Kynurenine-degrading enzymes, direct AhR antagonists, and tryptophan mimetics are advancing in early-stage or preclinical development. Ample preclinical evidence supports the continued development of commensal Trp–indole–AhR pathway inhibitors to augment efficacy of immune-checkpoint and other forms of therapy. Based on its immunosuppressive and cancer-promoting effects, Trp degradation remains an important target in immuno-oncology. In future clinical trials, it will be critical to stratify patients to inhibitors of Trp catabolism based on enzyme expression, Trp catabolite levels and the activation of downstream signalling pathways, as only those patients whose tumors indeed show expression and activity of Trp-catabolising enzymes can benefit of these therapies. Furthermore, strategies targeting AhR activity might be beneficial in tackling immunosuppression and malignant properties of tumor cells mediated by Trp catabolites.

## Figures and Tables

**Table 1:** AhR Endogenous Ligands.

AhR Ligand	Source	Function	Reference
Tryptamine	Microbiota	Decreases TH17	[79]
Indole-3-aldehyde	Microbiota	Increases IL-22	[30]
Indole-3-acetic acid	Microbiota	Induces COX-2	[80]
Indole propionic acid	Microbiota	Suppresses CNS and Gut inflammation	[28,81]
Indoxyl-3-sulfate	Microbiota/Host cells	Suppresses CNS Inflammation	[63]
Skatole	Microbiota	IEC death	[82]
l-kynurenine	Host cells	Antimicrobial activites, increases IDO1 and TGF-β	[76,83]
Kynurenic acid	Host cells	Tumor cell migration	[84]
Xanthurenic acid	Host cells	Tumor cell migration	[84]
Cinnabarinic acid	Host cells	Increases IL-22	[85]
FICZ	Photo-oxidation	Enhances TH17 differentiation	[10]
Indirubin	Plant/Host cells	Increases CYP1A1	[86]
Indigo	Plant/Host cells	Protects against HFD-induced glucose dysregulation	[86,87,88]
ITE	Porcine Lung	Reduces IL-6 and TNF-α	[75]

## Data Availability

Data sharing is not applicable to this article.
